# Ultrasound measurements of carotid intima-media thickness and plaque in HIV-infected patients on the Mediterranean diet

**DOI:** 10.3325/cmj.2013.54.330

**Published:** 2013-08

**Authors:** Klaudija Višković, George W. Rutherford, Gabriel Sudario, Lorna Stemberger, Zoran Brnić, Josip Begovac

**Affiliations:** 1 Department of Radiology and Ultrasound, University Hospital for Infectious Diseases, Zagreb, Croatia; 2 Global Health Sciences, University of California, San Francisco, CA, USA; 3 Outpatient Center for HIV/AIDS, University Hospital for Infectious Diseases, Zagreb, Croatia; 4 University of Zagreb School of Medicine, Zagreb, Croatia

## Abstract

**Aim:**

To evaluate the influence of food habits, specifically adherence to the Mediterranean diet, on carotid intima-media thickness (CIMT) and the presence of plaques in HIV-infected patients taking antiretroviral therapy (ART) and non-HIV-infected participants and to determine if HIV infection contributes independently to subclinical atherosclerosis.

**Methods:**

We conducted a cross-sectional study of 110 HIV-infected patients on ART and 131 non-HIV-infected participants at the University Hospital for Infectious Diseases in Zagreb, Croatia, from 2009-2011. CIMT measurement and determination of carotid plaque presence was detected by ultrasound. Adherence to the Mediterranean diet was assessed by a 14-point food-item questionnaire. Subclinical atherosclerosis was defined by CIMT≥0.9 mm or ≥1 carotid plaque.

**Results:**

In HIV-infected patients, subclinical atherosclerosis was associated with older age (*P* < 0.001; Mann-Whitney test), higher body mass index (*P* = 0.051; Mann-Whitney test), hypertension (*P* < 0.001; χ^2^ test), and a lower Mediterranean diet score (*P* = 0.035; Mann-Whitney test), and in non-HIV-infected participants with older age (*P* < 0.001; Mann-Whitney test) and hypertension (*P* = 0.006; χ^2^ test). Multivariate analysis showed that decreased adherence to the Mediterranean diet was associated with higher odds of subclinical atherosclerosis (odds ratio [OR] 2.28, 95% confidence interval [CI] 1.10-4.72, *P* = 0.027) as was current smoking (OR 2.86, 95% CI 1.28-6.40), hypertension (OR 3.04, 95% CI 1.41-6.57), and male sex (OR 2.35, 95% CI 0.97-5.70). There was a significant interaction of age and HIV status, suggesting that older HIV-infected patients had higher odds of subclinical atherosclerosis than controls (OR 3.28, 95% CI 1.24-8.71, *P* = 0.017 at the age of 60 years).

**Conclusion:**

We confirmed the association between lower adherence to the Mediterranean diet and increased risk of subclinical atherosclerosis and found that treated HIV infection was a risk factor for subclinical atherosclerosis in older individuals.

The advent of antiretroviral therapy (ART) for treatment of human immunodeficiency virus (HIV) infection has improved the quality of life and life expectancy of HIV-infected patients (1,2). However, a number of comorbidities have emerged, including atherosclerosis (3,4).

The impact of HIV infection and exposure to ART on development of subclinical atherosclerosis is incompletely understood (1,5). Several factors may contribute to an increased risk of coronary and cerebrovascular artery disease in HIV-infected patients: chronic endothelial or myocardial inflammation due to HIV infection per se, dyslipidemia associated with ART, and the interaction of treatment with traditional risk factors for cardiovascular disease (smoking, hypertension, age, etc) (1). Also, several antiretroviral drugs are believed to be partially related to an increase in cholesterol levels (6). The risk and problems associated with ART have led to the study of treatment-sparing strategies, which might provide the benefits of ART while minimizing the risk of adverse advents and other risks (7). The Strategies for Management of Antiretroviral Therapy (SMART) trial was conducted to compare the episodic use of ART according to the CD4+ count with the current practice of continuous ART (7).

An early marker of atherosclerosis is an increase in carotid intima-media thickness (CIMT), which enables the risk assessment of coronary and cerebral-artery disease (8). Carotid plaque area is another marker, which is more strongly associated with traditional risk factors and is more predictive of myocardial infarction than of stroke (9,10). There are many studies on the association of HIV infection, HIV disease parameters, and ART use with subclinical carotid artery atherosclerosis, yet there is still controversy over whether chronic HIV infection and ART (mainly protease inhibitors, PI) contributes to subclinical atherosclerosis. Although there are cross-sectional studies reporting an association between HIV infection, ART, and subclinical atherosclerosis (11-16), a number of studies did not find such an association (1,17-19).

The Mediterranean diet (20) has been shown to protect against cardiovascular disease and other chronic conditions (21-23). However, only a few studies have investigated the relationship of dietary intake with body fat changes and metabolic abnormalities in HIV-infected patients (24-26). To the best of our knowledge, the association between the adherence to Mediterranean diet and CIMT and the presence of plaques in HIV-infected patients taking ART has never been explored.

The aims of the present study are to evaluate the influence of food habits, specifically adherence to the Mediterranean diet, on CIMT and the presence of plaques in HIV-infected patients taking ART and non-HIV-infected participants and to assess if HIV-infection contributes independently to subclinical atherosclerosis.

## Methods

### Study design

We conducted a cross-sectional study comparing CIMT and the presence of carotid plaques as surrogate markers of atherosclerosis in HIV-infected patients and non HIV-infected participants.

### Study population

We selected participants from a consecutive sample of HIV-infected patients who had been on ART for ≥12 months at the University Hospital for Infectious Diseases (UHID) in Zagreb, Croatia. Non HIV-infected participants were healthy volunteers who were UHID employees or individuals who came to the Department of Radiology and Ultrasound at UHID for routine preventive ultrasound check-ups. They were not tested for HIV before the study. Participants were consecutively recruited and enrolled over a 20-month period beginning in July 2009. Inclusion criteria for HIV-infected patients were age of over 18 years with documented HIV infection (enzyme immunoassay and Western-blot positive) and at least 12 months of ART, and for controls the age of 18 years or older. Exclusion criteria were dementia, current acute illness, current use of drugs that modify lipid and glucose metabolism (insulin, metformin, glibenclamide, glimepiride, glyquidone, acarbose, rosiglitozone, pioglitazone, sitagliptin, vildagliptin, repaglinide, exenatide, liraglutide, simvastatin, fluvastatin, lovastatin, atrovastatin, rosuvastatin, gemfibrozil, fenofibrate, ezetimibe, omega-3 acids), as well as diuretics and corticosteroids. We also excluded participants with a history of cardiovascular disease or stroke, documented hepatitis C infection, diabetes mellitus, and allergy or intolerance to olive oil, fish, or nuts. All participants provided written informed consent prior to enrolment. The study protocol was reviewed and approved by the Ethics Committee of UHID and the Committee on Human Research at the University of California, San Francisco.

### Ultrasound measurements and definitions of outcomes

Participants were enrolled and underwent ultrasound measures at the same visit. Measurements of CIMT and assessment of the plaque presence was performed by a single radiologist, who was blinded to the participants’ HIV status and Mediterranean diet score, using a linear array probe (10 MHz and 42 mm) in the supine position. We examined the left and right common carotid arteries in anterolateral, posterolateral, and mediolateral directions and obtained longitudinal images of the distal common carotid arteries in which the interfaces were most clear. CIMT was measured in the far wall of the common carotid artery, 1 cm proximal to the carotid bulb in the region free of plaques with the optimum angle for measuring at the proximal and distal wall (27).

We defined plaque as a focal wall thickening 50% greater than the surrounding wall thickness, confirmed during scanning by electronic calipers (28). We examined the left and right carotid bifurcations and internal and common carotid arteries for the presence of plaque. Subclinical atherosclerosis was defined as CIMT≥0.9 mm and/or the presence of ≥1 carotid plaque (10).

### Nutritional and clinical assessment

Prior to the ultrasound, each participant was asked to fill out a 14-point food-item questionnaire to assess the adherence to the traditional Mediterranean diet. The questionnaire was originally developed and validated by Babio et al (29,30) and was used to rapidly control for compliance with the dietary intervention of the Prevencion con Dieta Mediterranea (PREDIMED) study, a multicenter clinical trial aimed at assessing the effects of the traditional Mediterranean diet on the primary prevention of cardiovascular disease (31). It was translated into Croatian, and completed during a face-to-face interview with the attending physician. From the questionnaire we derived the composite dietary score, dichotomized it at the median, and defined low adherence to the Mediterranean diet as a score of <4. We predefined the use of the median because of our relatively small sample size.

We abstracted additional data for HIV-infected patients, including medication history, from medical records using a standardized abstraction form. Patients were considered as having been treated with a particular drug or drug class if the drug combination had been administered for more than 12 months. We measured the patients’ weight with a standard physician’s office balance scale and rounded the weight to the nearest 0.1 kg, and the height using a wall-mounted stadiometer and rounded the height to the nearest 0.1 cm. Blood pressure measurements were obtained in a seated position (at the right upper arm), using a standard mercury sphygmomanometer, expressed in terms of the systolic pressure over diastolic pressure in millimeters of mercury (mmHg). Hypertension was defined according to the guidelines of the Seventh Report of the Joint National Committee on Prevention, Detection, Evaluation and Treatment of High Blood Pressure, that is, systolic blood pressure ≥140mmHg or diastolic blood pressure ≥90mmHg and/or concomitant use of antihypertensive medications (32).

We categorized the cigarette smoking status as current or non-current smoker. Previous cardiovascular diseases (as an exclusion criterion) included history of angina, myocardial infarction, coronary artery disease, atrial fibrillation, congestive heart failure, and valvular heart disease (28). Stroke was defined by the National Institute of Neurological Disorders and Stroke Classification of Cerebrovascular Diseases III (28).

We determined plasma HIV viral load using COBAS Amplicor HIV-1 Monitor Test, version 1.5 (Roche Diagnostic Systems, Basel, Switzerland) with a lower limit detection of 50 copies per milliliter (ultrasensitive method). Absolute CD4 T-cell counts (in cells/µL) were measured using Flow Count Fluorospheres (Beckman Coulter, Fullerton, CA, USA) and four-color flow cytometry was performed using Cytomics FC500 flow cytometer (Beckman Coulter, Fullerton, CA, USA).

### Statistical analysis

. Categorical variables are presented as proportions and non-normally distributed continuous variables as medians and interquartile ranges (IQR). The normality of distribution was assessed graphically and by the Shapiro-Wilk test. To compare different characteristics of participants with and without subclinical atherosclerosis, we used the Whitney-Mann test for continuous variables and Fisher exact test or χ^2^ test for categorical variables, separately for HIV-infected participants and non HIV-infected participants. The odds of having subclinical atherosclerosis were calculated in crude logistic regression models, which included one independent variable (body mass index [BMI], sex, smoking, hypertension, Mediterranean diet score, or HIV status) and age and their interaction for the whole study population (N = 241). In multivariate logistic regression model analysis, we included all the above mentioned variables except BMI (non-significant in crude analysis) and the interaction of age with HIV status. Statistical analyses were performed with SAS software, version 9.1.3. (SAS Institute, Cary, NC, USA). The level of statistical significance was set at *P* < 0.05.

## Results

We enrolled 110 HIV-infected patients and 131 non-HIV-infected participants. All the participants were Caucasian. HIV-infected patients were more frequently male, younger, had a lower BMI, and a higher Mediterranean diet score (Table 1).

**Table 1:** Characteristics of HIV-infected (n = 110) and non-HIV-infected participants (n = 131) examined by ultrasound to detect subclinical atherosclerosis

	HIV-infected patients (n = 110)	non HIV-infected patients (n = 131)	*P*
Male sex (n, %)	93 (84.5)	91 (69.5)	0.006*
Age, years (median, range^‡^)	46.5 (40-56)	52 (45-61)	0.003†
Weight, kg (median, range)	78 (70-84)	85 (73-98)	<0.001†
Body mass index, kg/m^2^ (median, range)	24.8 (22.5-26.4)	27.8 (24.5-30.8)	<0.001†
Mediterranean diet score (median, range)	5 (3-7)	3 (2-5)	<0.001†
Carotid intima-media thickness, right, in millimeters (median, range)	0.67 (0.53-0.82)	0.68 (0.61-0.82)	0.640†
Carotid intima-media thickness, left, in millimeters (median, range)	0.68 (0.61-0.83)	0.72 (0.58-0.83)	0.591†
Systolic blood pressure in mmHg (median, range)	130 (120-140)	130 (120-140)	0.436†
Participants with hypertension (n, %)	31 (28.1)	29 (22.1)	0.240*
Current smoker (n, %)	40 (36.4)	30 (22.9)	0.022*
Carotid intima-media thickness ≥0.9 mm (n, %)	31 (28.2)	25 (19.1)	0.095*
Presence of ≥1 carotid plaque (n, %)	26 (23.6)	31 (23.7)	0.996*
Presence of ultrasound-assessed subclinical atherosclerosis (n, %)	37 (33.6)	42 (32.1)	0.795*

*χ^2^ test.

†Mann-Whitney test.

More than 50% of the 110 HIV-infected patients were on non-nucleoside reverse transcriptase inhibitors-containing antiretroviral regimens (Table 2). Of 110 patients, 106 (96.4%) had an undetectable viral load (<50 copies per milliliter). Of 4 patients with detectable viral load, one had discontinued antiretroviral treatment and three had low-level viremia (<1000 copies/mL). HIV-infected participants taking PI and those taking other antiretroviral regimens showed no differences in ultrasound-measured CIMT medians, CIMT≥0.9 mm, and ≥1 carotid plaque presence (*P* = 0.819, *P* = 0.791, and *P* = 0.904, respectively).

**Table 2:** Duration of antiretroviral therapy (ART), currently used ART, current plasma HIV-1 viral load, and CD4 cell counts in HIV-infected patients (n = 110) at the time of ultrasound measurement of carotid intima-media thickness (CIMT) and the presence of plaque

Duration of ART in years (n, %)	5.1 (2.6-8.4)
Duration of exposure to protease inhibitors (years) (n, %)	3.8 (1.5-6.5)
ART combination (n, %):	
two nucleoside or nucleotide reverse transcriptase inhibitors plus one non-nucleoside reverse transcriptase inhibitor	73 (66.4)
two nucleoside or nucleotide reverse transcriptase inhibitors plus one protease inhibitor	28 (25.5)
other, including entry inhibitor or integrase inhibitor	9 (8.1)
Current CD4 cell count in cells/µL (median, range)	467 (311-584)
HIV RNA≥50 copies/mL (n, %)	4 (3.6)

Subclinical atherosclerosis was found in 79 (32.6%) of 241 participants (Table 3): 37 (33.6%) of 110 HIV-infected patients and 42 (32.1%) of 131 non HIV-infected participants. The proportion of men and women in HIV-infected and non-HIV-infected group with subclinical atherosclerosis was not different (*P* = 0.08 and *P* = 0.632, respectively). In HIV-infected patients, subclinical atherosclerosis was associated with older age (*P* < 0.001, Mann-Whitney test), higher BMI (*P* = 0.051; Mann-Whitney test), and hypertension (*P* < 0.001; χ^2^ test). In non HIV-infected participants, subclinical atherosclerosis was associated with older age (*P* < 0.001; Mann-Whitney test) and hypertension (*P* = 0.006; χ^2^ test). Patients with subclinical atherosclerosis had lower median Mediterranean diet scores. In non HIV-infected participants, 24 of 42 (57%) with subclinical atherosclerosis and 42 of 89 (47%) without subclinical atherosclerosis had a Mediterranean diet score <4 (*P* = 0.288; χ^2^ test). In HIV-infected participants, 17 of 37 (46%) with subclinical atherosclerosis and 18 of 73 (25%) without subclinical atherosclerosis had a Mediterranean diet score <4 (*P* = 0.024; χ^2^ test). Among participants >45 years old, more HIV-infected patients than non-HIV-infected participants had subclinical atherosclerosis (56% vs 40%, *P* = 0.042).

**Table 3:** Ultrasound-assessed subclinical atherosclerosis of HIV-infected (n = 110) and non-HIV-infected participants (n = 131) by demographic characteristics and adherence to Mediterranean diet scores

Variable	HIV-infected patients with subclinical atherosclerosis(n = 110)		*P*	Non HIV-infected participants with subclinical atherosclerosis (n = 131)		*P*
	yes (n = 37)	no (n = 73)		yes (n = 42)	no (n = 89)	
Age in years (median, range)^‡^	58 (53-65)	42 (37-47)	<0.001*	59.5 (52-65)	50 (42-56)	<0.001*
Body mass index (median, range)	25.3 (24.3-26.4)	24 (22.1-26.4)	0.051*	28.1 (24.5-32.1)	27.7 (24.5-30.4)	0.407*
Systolic blood pressure in mmHg (median, range)	140 (120-150)	120 (115-130)	<0.001*	140 (130-150)	125(120-135)	<0.001*
Hypertension (n, %)	19 (51.4)	13 (17.9)	<0.001†	15 (35.7)	13 (14.6)	0.006†
Mediterranean diet score (median, range)	4 (3-6)	6 (4-7)	0.035*	3 (3-5)	4 (2-5)	0.692*

*Mann-Whitney test.

†χ^2^ test.

**Figure 1:**
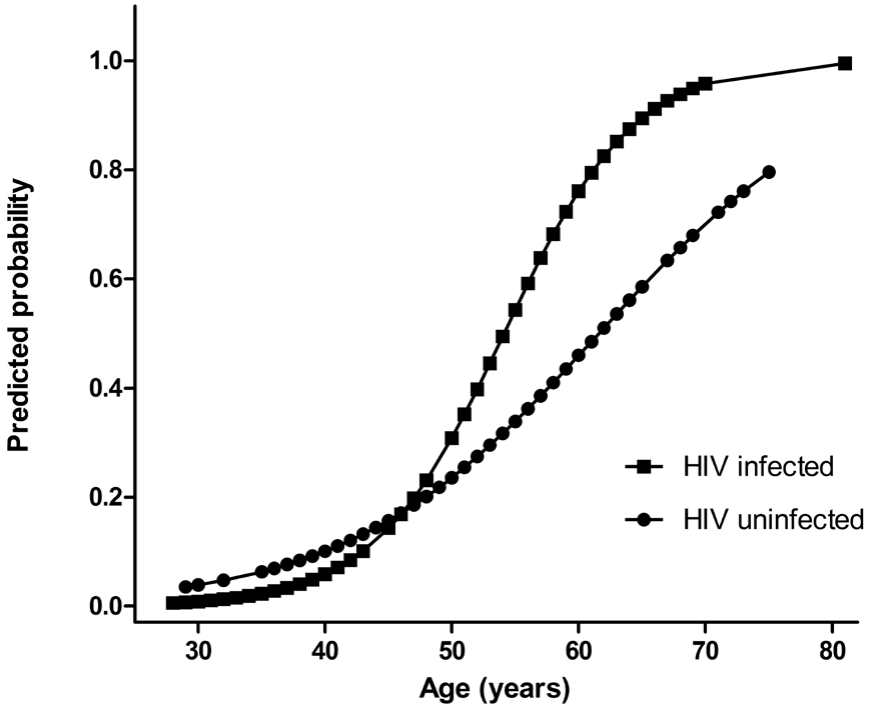
Estimated probability of subclinical atherosclerosis according to HIV status and age

When the whole study population was analyzed using one independent variable and age in the crude analysis model, subclinical atherosclerosis was associated with current smoking, male sex, and hypertension (Table 4). In multivariate analysis, subclinical atherosclerosis remained associated with all risk factors including lower adherence to the Mediterranean diet (Mediterranean diet score <4) (odds ratio 2.28; 95% confidence interval 1.10-4.72; *P* = 0.027) (Table 4). There was a significant interaction of age and HIV status in both crude (*P* = 0.028) and multivariate analysis (*P* = 0.029), suggesting that the probability of having subclinical atherosclerosis increased in HIV-infected patients after the age of 45 years more rapidly than in non-HIV-infected individuals (Figure 1).

**Table 4:** Crude and adjusted odds of subclinical atherosclerosis by patient characteristics and Mediterranean diet score in HIV-infected (n = 110) and non-HIV-infected (n = 131) participants

	Crude analysis*		Multivariate analysis^†^	
	Odds ratio (95% confidence interval)	*P*	Odds ratio (95% confidence interval)	*P*
Mediterranean diet Score:				
<4	1.83 (0.96-3.48)	0.065	2.28 (1.10-4.72)	0.027
≥4	1		1	
Current smoker:				
yes	2.65 (1.26-5.54)	0.010	2.86 (1.28-6.40)	0.010
no	1		1	
Sex:				
male	2.80 (1.23-6.36)	0.014	2.35 (0.97-5.70)	0.058
female	1		1	
HIV- status at the age of 60 y:				
HIV-infected	3.74 (1.50-9.31)	0.005	3.28 (1.24-8.71)	0.017
HIV non-infected	1		1	
Hypertension:				
yes	3.72 (1.81-7.67)	<0.001	3.04 (1.41-6.57)	0.005
no	1		1	

*Adjusted for age and one characteristic.

†Adjusted for age, Mediterranean Diet Score, current smoking status, sex, HIV-status, and hypertension.

## Discussion

We found that subclinical atherosclerosis was common both among HIV-infected and non-HIV-infected participants and was associated with older age, male sex, current smoking, and hypertension. Lower Mediterranean diet scores were associated with higher odds of subclinical atherosclerosis. Older HIV-infected patients treated with ART were more likely to have subclinical atherosclerosis than non HIV-infected individuals.

Croatians living near the Adriatic Sea have a relatively lower occurrence of chronic diseases such as cancer, diabetes, obesity, and cardiovascular diseases than Croatians living in the continental area, which has been linked to adherence to the Mediterranean diet (33). Although it is hard to define what exactly constitutes the Mediterranean diet as there are as many Mediterranean eating patterns as there are Mediterranean countries (33), the main staples always include olive oil, fish, and red wine.

The questionnaire used in our study was a slightly modified questionnaire previously validated in Spain, which is suitable to assess the nutritional habits of the Croatian population living in the Mediterranean region (29,34,35). This brief 14-item questionnaire is less time-demanding, less expensive, and requires less collaboration from participants than the usual full-length food frequency questionnaires or other more comprehensive methods (31). In addition, it allows the possibility of providing feedback to the participant immediately upon completion. It was the key element in the PREDIMED trial and had been previously validated against food frequency questionnaires used in the study (23). Full-length food frequency questionnaires usually used 100 items, 24-hour recalls, or other time-consuming methods, to evaluate adherence to the Mediterranean dietary pattern. In contrast to these methods, the brief tool assessing only a small number of foods measured in servings/d or servings/week enables physicians and participants to negotiate changes in patients’ dietary quality and set goals in easily understandable units (food servings) (31).

In a previous observational study conducted in Croatia, no association was found between plasma lipid changes during the first year of ART and adherence to the Mediterranean diet (20). This association was also not observed in non-HIV-infected populations (36). In the multivariate model of our study, low adherence to the Mediterranean diet was, however, significantly associated with subclinical atherosclerosis. It is possible that there is a synergy among the nutrient-rich components included in the Mediterranean diet that fosters favorable changes in the intermediate pathways of cardiometabolic risk (23).

To determine cardiovascular risk among HIV-infected persons, we used surrogate markers of subclinical atherosclerosis: ultrasound-measured CIMT and the presence of ≥1 plaque. Higher levels of CIMT have been found to predict serious adverse cardiovascular events (30). Greater CIMT than in controls (0.004 mm) has also been associated with HIV infection in a meta-analysis of 13 cross-sectional studies (37,38). However, a significant difference in CIMT and plaque prevalence between HIV-infected patients and non-HIV infected participants has not been found (1,18,19). One of the possible explanations for controversial results is the limited ability of anatomic imaging to comprehensively define the cardiovascular risk (38). We found more subclinical atherosclerosis cases in HIV-infected patients than in non HIV-infected controls after the age of 45 years.

The SMART trial found a higher risk of cardiovascular events among patients with discontinuation of ART than in patients with uninterrupted ART (7). This suggests an impact of HIV infection on cardiovascular events, possibly mediated through inflammation and thrombosis induced by HIV (39).

It is quite possible that traditional cardiovascular risk factors are more important predictors of atherosclerosis than measurable HIV-related factors. In our HIV-infected participants, the factors significantly associated with subclinical atherosclerosis were smoking and hypertension. Smoking is common in HIV-infected patients and the Data Collection on Adverse events of Anti-HIV Drugs study found that smoking was the most powerful predictor of cardiovascular diseases (40).

Previous studies have suggested that there may be an association between the use of PI and increased CIMT (30,41). One quarter of HIV-infected participants in our study were on PI regimens for at least 12 months. We did not find any difference in surrogate markers of atherosclerosis between patients who were on a PI regimen and those who were not. However, we cannot rule out this possibility due to the small sample size. Therefore, a longitudinal follow-up on our participants is planned to determine if HIV infection or PI therapy is important in the progression of subclinical atherosclerosis.

Our study has some important limitations. First, we utilized a cross-sectional design to determine the risk factors, CIMT, and plaque levels. Given the fact that the effects of HIV and ART on cardiovascular risk factors, such as cholesterol, high-density lipoproteins, and blood pressure are dynamic and cumulative, a longitudinal follow up would be better suited. Second, we did not measure biochemical correlates of atherosclerosis, such as total cholesterol, high-density lipoproteins and low-density lipoproteins, which could potentially have provided additional evidence of the protective effect of the Mediterranean diet. Finally, non HIV-infected participants should ideally have been identical to infected patients except for the presence of HIV-infection, but given the highly specialized nature of our institution, it was not possible to precisely match infected and non-infected patients.

Our study demonstrated an association of subclinical atherosclerosis with specific and traditional dietary habits, which had been associated with decreased all-cause mortality and improvement in cardiovascular risk factors in prior studies in the general population. We also provided evidence that HIV infection treated with ART was associated with accelerated atherosclerosis in older individuals. These results should be considered in clinical assessment of cardiovascular risk in HIV-infected patients.
